# Response: Commentary: High-intensity Intermittent Training vs. Moderate-intensity Intermittent Training: Is It a Matter of Intensity or Intermittent Efforts?

**DOI:** 10.3389/fphys.2017.00526

**Published:** 2017-07-21

**Authors:** David Jiménez-Pavón, Carl J. Lavie

**Affiliations:** ^1^Galeno Research Group, Department of Physical Education, Faculty of Education Sciences, University of Cádiz Cádiz, Spain; ^2^Department of Cardiovascular Diseases, John Ochsner Heart and Vascular Institute, Ochsner Clinical School, Medicine Program, The University of Queensland New Orleans, LA, United States

**Keywords:** high intensity interval training, moderate intensity interval training, periodization, health

We have read the Commentary by Gentil and Del Vecchio (2017), and we are grateful for the interest shown. Exactly, the main objective of our article was to highlight the relevance of prescribing and researching on moderate-intensity interval training (MIIT), although not only this, but in parallel with the current research on high-intensity interval training (HIIT). In addition, we highlight the necessity of considering all of the methodological differences between two training regimens (intensity, type of stimulus, rest period, training density, etc.) before making conclusions based only on one of these parameters. Otherwise, it would be hard to compare HIIT vs. aerobic continuous training under the assumption that the unique difference is intensity. Then, we cited three different studies showing results that support the following heading from our article “‘*Intensity’ is not the only difference between HIIT and continuous training*.” By contrast, we did not suggest that all the results are due to intermittent stimulus rather than intensity, in fact, we literally wrote “*We speculate that some of the effects could be achieved with both (MIIT and HIIT), while maybe others would be achieved only using HIIT.”*

Gentil and Del Vecchio (2017) mentioned that the study performed by (Rakobowchuk et al., 2012) cannot be considered a comparison between HIIT and MIIT due to the fact they trained two groups at the same intensity (120% of the peak work rate obtained in a ramp-incremental test). However, these authors state in their introduction how to manage the exercise intensity by modulating the interval duration, with short-duration intervals displaying a lower metabolic stress than longer duration intervals despite the power output, training duration, and total work being similar between the two (Turner et al., 2006). They also explain that MIIT intervals were chosen to maintain a metabolic rate below lactate threshold, while the HIIT intervals would induce a raised, but steady-state, blood lactate response (Turner et al., 2006). Because of that, we cited this study as an example of how managing with different parameters of a training model the exercise intensity can be affected, and consequently the results could be different. In addition, Gentil and Del Vecchio (2017) suggest that the study conducted by Alkahtani et al. (2013) cannot be used to make inferences about training intensity as the other parameters were not equal. We agree they use different interval durations which could affect the results, but we did not cite these papers as the best option or methodological design to compare HIIT and MIIT, and these are not the only works addressing this comparison or research question. In fact, we highlight the necessity of more well-designed research aimed to analyze the differences on physiological and health aspects depending of the combination of many training parameters, including intensity.

Regarding the aspect commented on the study by Racil et al. (2013), we agree that it is the closest approach to an appropriate design locking at the different effect of HIIT and MIIT. However, we would like to clarify again that we do not state intermittent nature as more relevant than intensity in achieving results, but we highlight the necessity to consider also that (and many other parameters) rather than just intensity. Moreover, based on the comments made by Gentil and Del Vecchio (2017), we consider that they may agree with us that to compare HIIT vs. aerobic continuous exercise is not necessarily more appropriate.

We agree that defining HIIT intensity is not a matter of “the more the better” (Gentil and Del Vecchio, 2017), thus, we suggest that defining an adequate intensity should consider that moderate to high (60–80%) and high (>85%) intensities could have different but important benefits on many health parameters, as well as the fact that both intensities are complementary in a well-designed and individualized periodization.

In line with the comment by Gentil and Del Vecchio (2017) that enjoyment of HIIT increased with training, whereas enjoyment of aerobic continuous training remained constant but lower. Again, it is difficult to know if this effect on enjoyment is due to intensity or by contrast due to continuous training mode which is known to be demotivating among beginners. Moreover, we really believe that it is true that enjoyment of HIIT increased with training, because of that we suggest that using MIIT in a first stage before including HIIT could help create an earlier appearance of enjoyment, however, as previously mentioned, there is a lack of research in this area to made a full comparison of HIIT and MIIT.

Finally, the limitations attributed to the references presented are the limitations of the current existing literature on this topic, and this should be considered in future research in order to cover all the gaps and methodological aspects mentioned. However, we reaffirm the relevance of using and researching not only on HIIT but also on MIIT. Moreover, studies comparing HIIT vs. aerobic continuous training should acknowledge the limitation that differences in the training model could also be critical.

## Author contributions

DJ lead the idea of the original paper and the response to the commentary and revise the full version. CL is the co-author working together with DJ in the developing and discussion of the rationale of the original paper and the current response to the commentary.

### Conflict of interest statement

The authors declare that the research was conducted in the absence of any commercial or financial relationships that could be construed as a potential conflict of interest.
